# Supplementary figures and images for: A comparison of humans and baboons suggests germline mutation rates do not track cell divisions
Source: PLoS Biol. 2020 Aug 17;18(8):e3000838. doi: 10.1371/journal.pbio.3000838 (PMC7467331; doi:10.1371/journal.pbio.3000838)

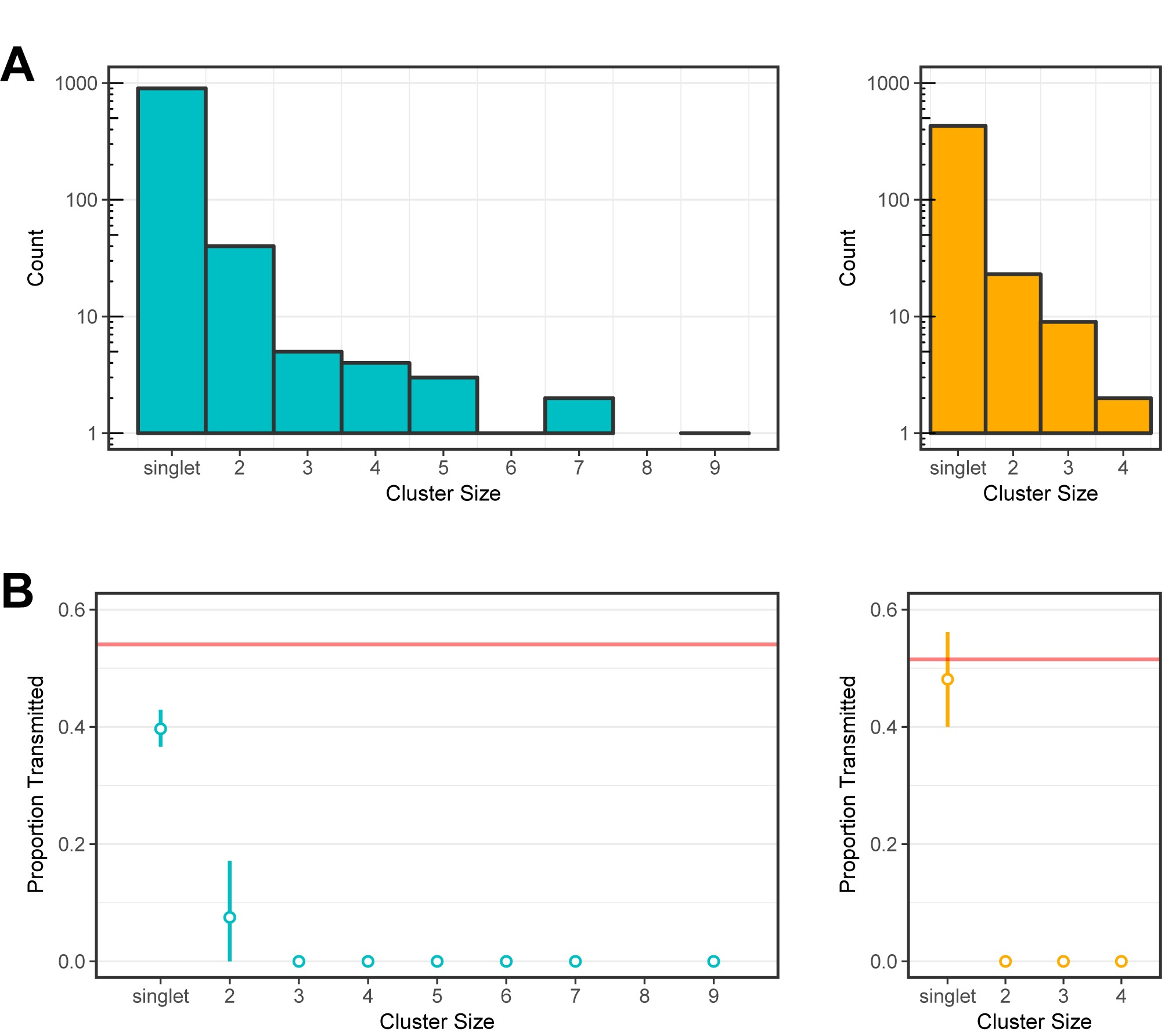

Supplement: S1 Fig — (A) Distribution of DNM cluster sizes (defined as the number of DNM calls within 100 bp of each other) in humans (left, teal) and baboons (right, orange). The height of each bar denotes the number of DNM clusters of the given size, as labeled on the x-axis. (B) Proportion of DNM clusters observed transmitted to the F2 generation, by size, in humans (left, teal) and baboons (right, orange). For each species, the horizontal red line denotes the mean proportion of DNMs expected to be observed in the F2 generation if called in the F1, as determined from simulations (see Methods). Vertical lines denote the 95% CI, as determined by bootstrap resampling clusters by 50 cM blocks [58,81,82]. Underlying data for this figure can be found in S2 Data. cM, centimorgan; DNM, de novo mutation. (TIF) [file pbio.3000838.s001.tif]

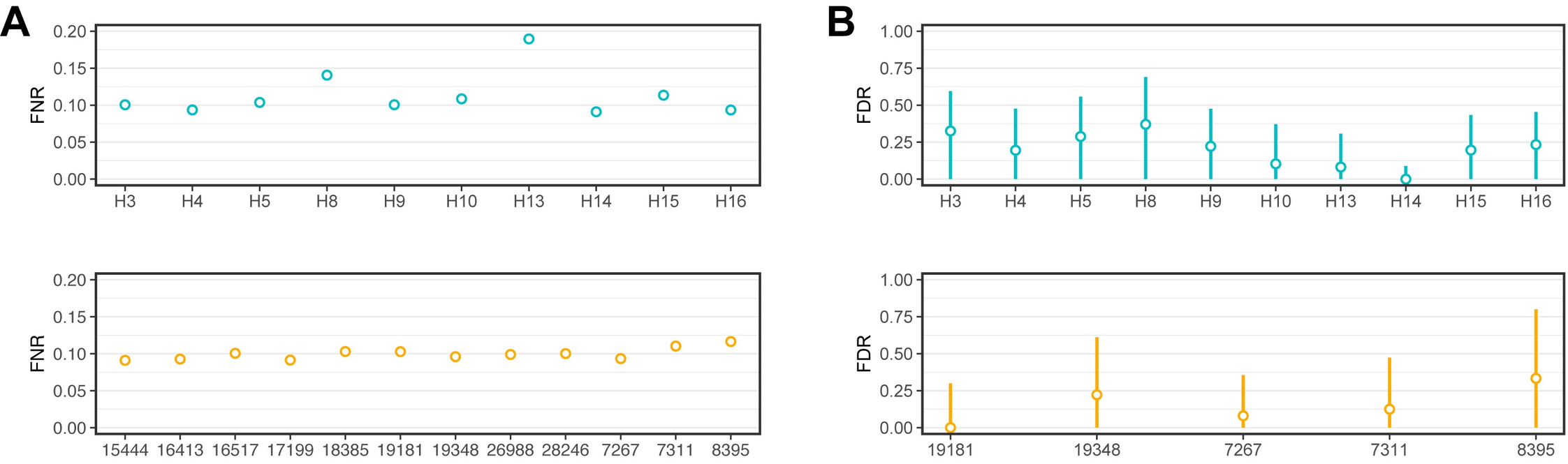

Supplement: S2 Fig — FNRs (in A) and FDRs (in B) of the DNM-calling pipeline estimated for each trio across humans (top, teal) and baboons (bottom, orange). In all plots, sample identifiers of the focal F1 are provided as labels on the x-axis. FNRs were estimated by applying the filtering pipeline to a set of simulated DNMs (20,000 per trio). For FNRs, 95% CIs from bootstrap resampling of mutations are narrow and hidden by the points. FDRs were inferred using a transmission-based approach in which the proportion of DNMs called in the F1 that were also observed in the F2 was compared against an expected proportion determined from simulations. Only the 5 baboon F1 individuals with at least 1 sequenced F2 offspring are depicted. Vertical lines denote 95% CIs from bootstrap resampling of 50 cM blocks [58,81,82]. See Methods for details on error rate estimation. Underlying data for this figure can be found in S2 Data. cM, centimorgan; DNM, de novo mutation; FDR, false discovery rate; FNR, false negative rate. (TIF) [file pbio.3000838.s002.tif]

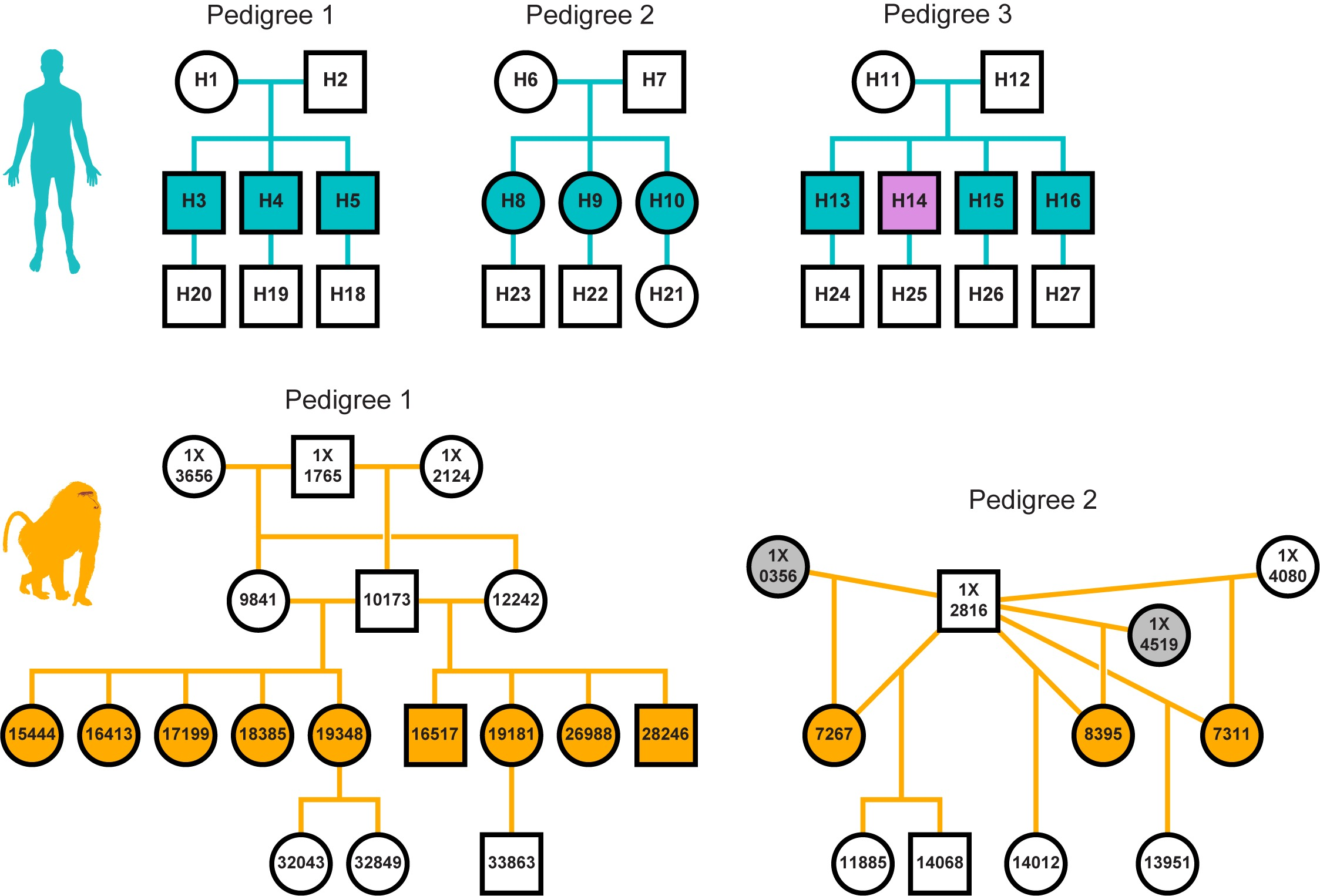

Supplement: S3 Fig — Reproduction of Fig 1 with unique sample identifiers for each individual in the human (top row, teal) and baboon (bottom row, orange) pedigrees. Sanger sequencing validation experiments focused on putative DNMs called in F1 individual H14, highlighted in lavender. Baboon mothers 1X0356 and 1X4519, highlighted in gray, lacked birth dates and were thus excluded from age effect analyses. DNM, de novo mutation. (TIF) [file pbio.3000838.s003.tif]

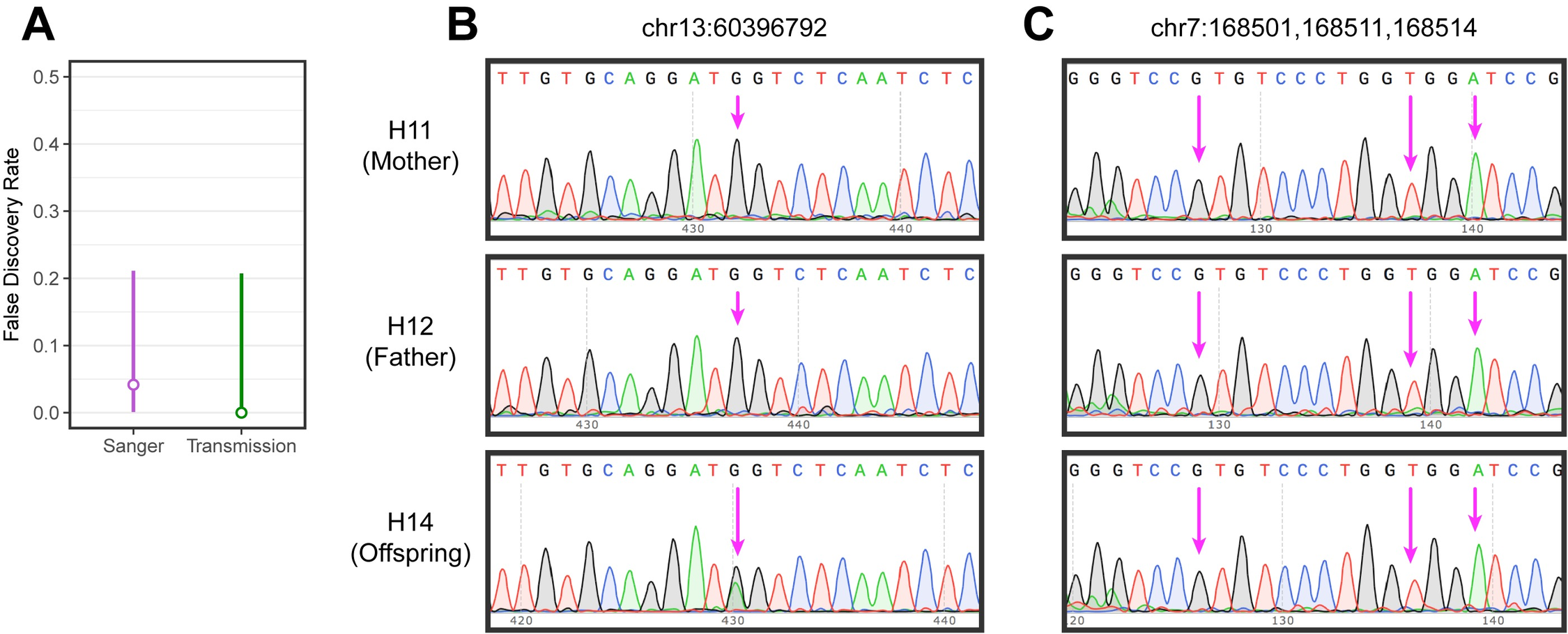

Supplement: S4 Fig — (A) Comparison of 2 methods for inferring the FDR of DNMs in human F1 individual H14. In one, the FDR was inferred by Sanger resequencing of putative DNMs (purple) and in the second, by a transmission-based approach (green) in which the number of DNMs observed in the F2 offspring of H14 was compared to the expectation determined by simulations (see main text). Vertical lines indicate the 95% CI of the point estimates. For the Sanger data, the binomial CI is indicated; for the transmission-based method, bootstrap resampling of 50 cM blocks [81,82] was used to obtain the interval. Examples of 4-color chromatograms from Sanger resequencing of a genuine G/C>A/T singlet DNM (in B) and a cluster of 3 spurious DNMs (in C). Chromatograms for the mother (top row) and father (middle row) of H14 (bottom row) are provided. Magenta arrows indicate the positions of putative DNMs originally called from Illumina sequence alignments. Absolute positions of the DNMs are also given at the top of each column using hs37d5 reference genome coordinates. Note the overlapping G (black) and A (green) peaks at the genuine DNM site in H14, indicating a heterozygous AG genotype, and the absence of a similar signal at the clustered calls. Underlying data for this figure can be found in S2 Data. cM, centimorgan; DNM, de novo mutation; FDR, false discovery rate. (TIF) [file pbio.3000838.s004.tif]

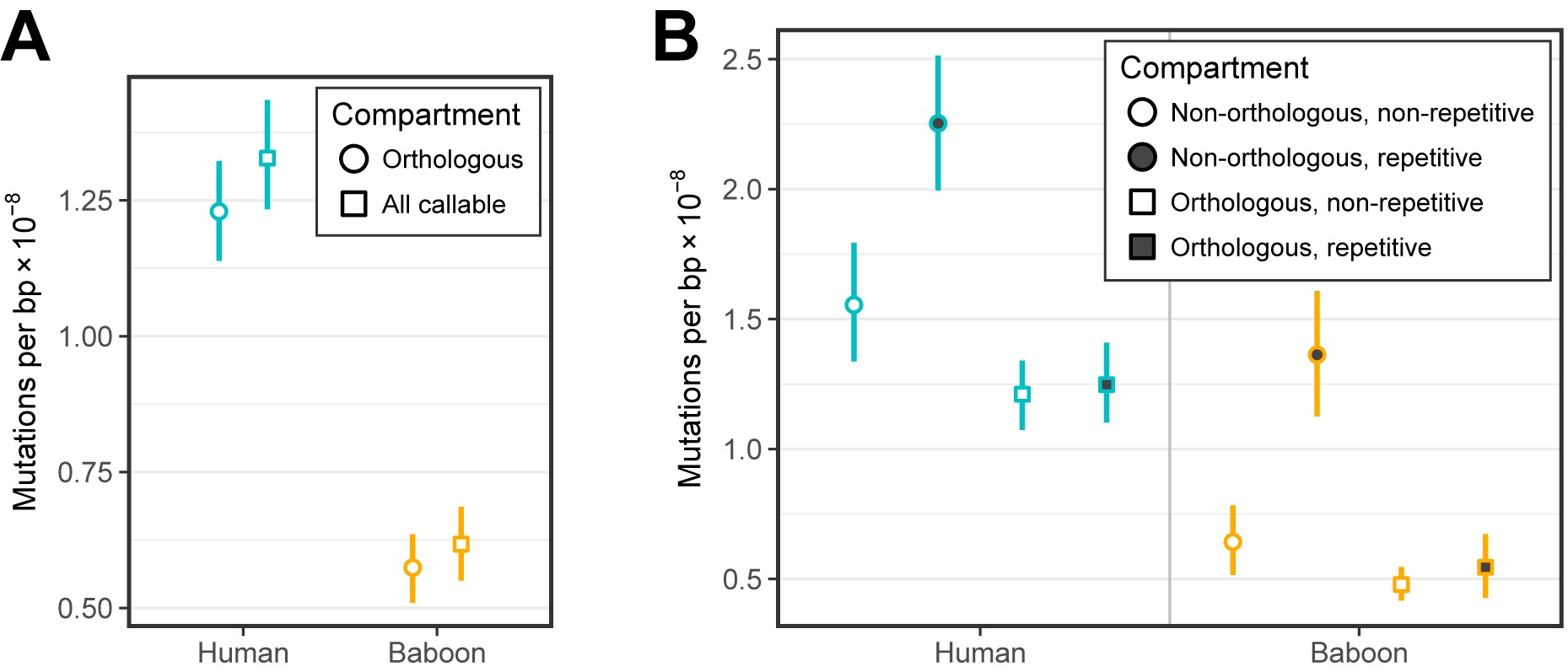

Supplement: S5 Fig — Human (teal) and baboon (orange) mutation rates were calculated across various subsets (compartments) of the genome. (A) Estimated mutation rates within and outside of regions orthologous between baboons and humans. Circles denote rates calculated using mutations identified within orthologous regions between the 2 species, whereas squares represent estimates calculated using all callable regions of the genome. The 95% CI shown around the estimates was obtained by resampling from the Poisson regression (see Methods). (B) Estimated mutation rates in compartments of the genome defined by their orthologous and repetitive status. Mutations were assigned to 4 mutually exclusive compartments based on whether they arose within or outside of orthologous and/or repetitive regions of the callable genome. For each compartment, mutation rates per generation were estimated by combining information across trios (see Methods). Compartments are nonorthologous and nonrepetitive (empty circles), nonorthologous and repetitive (filled circles), orthologous and nonrepetitive (empty squares), and orthologous and repetitive (filled squares). Vertical lines represent 95% CIs calculated by bootstrap resampling of 50 cM windows within individuals followed by aggregation of rates across individuals [58,81,82]. Underlying data for this figure can be found in S2 Data. cM, centimorgan. (TIF) [file pbio.3000838.s005.tif]

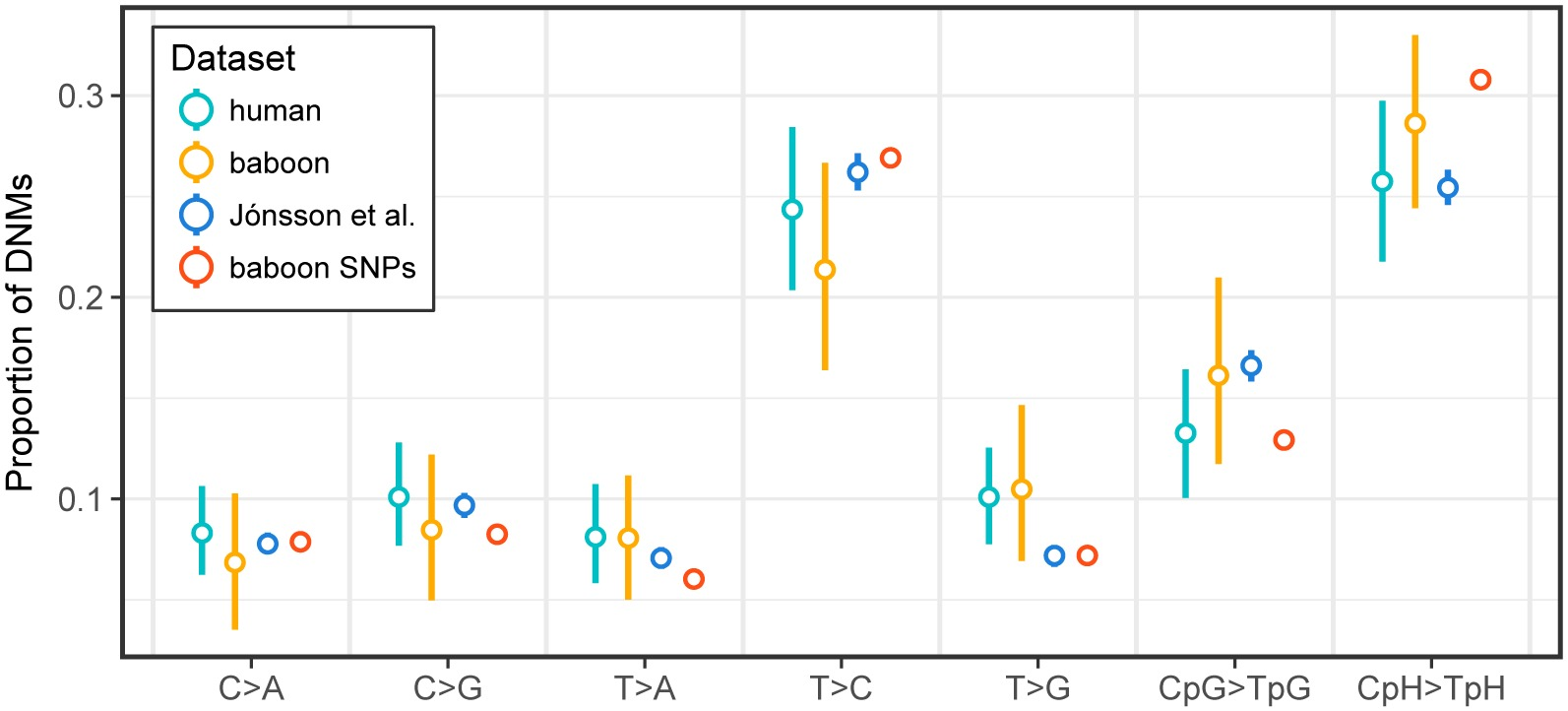

Supplement: S6 Fig — Spectra are shown for all DNMs (both transmitted and untransmitted to F2s) called in humans (teal); all baboon DNMs (orange); DNMs called by Jónsson and colleagues [17] in a large set of 3-generation pedigrees (blue); and low-frequency SNPs (doubleton and tripleton alleles) identified in a sample of 86 unrelated baboons (red). As in Fig 2C, each point indicates the relative proportion of each of 7 mutation types, as indicated on the x-axis. Reverse complement mutation types were collapsed together into a single type, and transitions at cytosine sites were split into those that occurred inside (CpG>TpG) or outside (CpH>TpH) of a CpG context. For all datasets, only DNMs and SNPs located within genomic regions classified as orthologous between humans and baboons were included. Vertical lines denote 95% CIs from bootstrap resampling of 50 cM blocks. CIs for estimates from the 2 reference datasets (blue and red) are small and largely hidden by the points. In humans, the proportion of T>G mutations differs significantly from the dataset by Jónsson and colleagues (forward variable selection p-value = 0.019); no other mutation types differed significantly in their proportions from the values in the reference set in either species (Jónsson and colleagues and the SNP panel, respectively). We note that given the inclusion of all mutation calls in F1s, regardless of whether they were transmitted to the F2, we expect some spurious calls to have been included in our de novo sets, more so than in the stringent set of DNMs shown in Fig 2C. Underlying data for this figure can be found in S2 Data. cM, centimorgan; CpG, 5′-cytosine-phosphate-guanine-3′; DNM, de novo mutation; SNP, single-nucleotide polymorphism. (TIF) [file pbio.3000838.s006.tif]

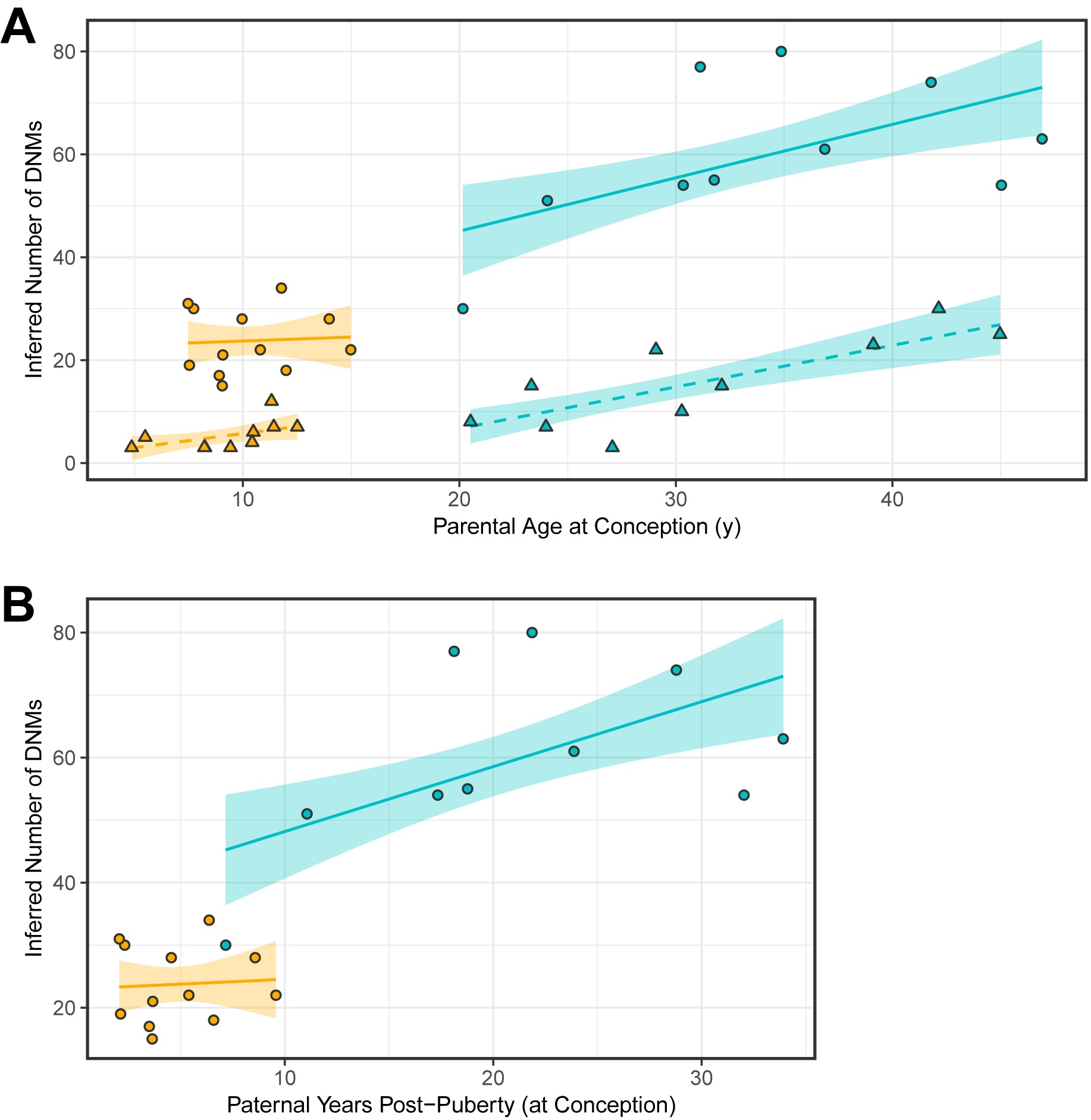

Supplement: S7 Fig — (A) Combined display of Fig 2A and Fig 2B showing human (teal elements) and baboon (orange elements) estimated DNM counts (y-axis) versus parental age at conception (x-axis) together in a single plot. Each F1 individual is represented by both a circular and triangular point denoting the number of mutations arising on the paternal or maternal genomes, respectively. Maternal points for 2 baboons were omitted because of a lack of data on maternal age. Solid and dashed lines indicate the Poisson regression maximum likelihood fit for males and females in each species. Shaded regions denote the 95% CIs on the regression coefficients. We failed to reject a model in which the paternal age effects of humans and baboons are the same (LR test p-value = 0.19). However, we find tentative evidence that the slope in baboons differs from the one estimated for humans based on a much larger data set by Gao and colleagues [32] (LR test p-value = 0.046). (B) Estimated DNM counts from fathers versus number of years between puberty and conception. Colors and elements are as in (A). Under a model in which paternal mutations track cell divisions post-puberty and the number of cell divisions and per-cell division mutation rates are the same in the 2 species, we would expect a higher baboon paternal age effect and similar intercept in the 2 species (see main text). For each species, the solid line denotes the best fit obtained from a Poisson regression of estimated paternal DNM count on paternal years between puberty and conception (x-axis), where puberty was assumed to occur at 13 years in humans and 5.41 years in baboons [33,35]. We do not reject a model in which the age effect is the same between species (LR test p-value = 0.19). A model in which human and baboon fathers accrue the same number of DNMs by puberty in males is somewhat unlikely (LR test p-value = 0.047) and a model in which both the paternal intercept and age effect parameters are the same in the 2 species even more so (LR t [file pbio.3000838.s007.tif]

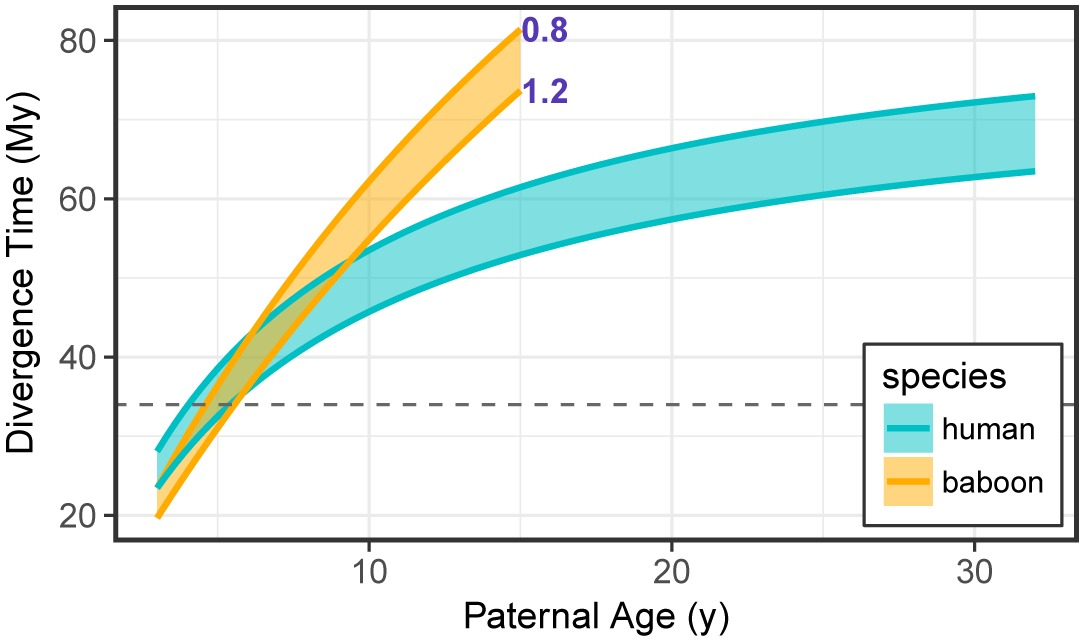

Supplement: S8 Fig — Divergence times were predicted using mutation and substitution rates measured in humans (teal) and baboons (orange), across a span of historical generation times. Human mutation rates were inferred using age effect parameters produced by Gao and colleagues [32], which were estimated from much more data (and are thus more precise). For this figure, as opposed to Fig 4D, baboon mutation rates were calculated using the parameters estimated in this study. Shaded areas cover the divergence times inferred across a span of plausible paternal generation times (x-axis) and paternal-to-maternal generation time ratios (ranging from 0.8 to 1.2, indicated in purple). The dashed gray line marks a plausible upper bound for the split time inferred from the fossil record [49,50]. As can be seen, the qualitative conclusion is the same as in Fig 4D. Underlying data for this figure can be found in S2 Data. OWM, Old World monkey. (TIF) [file pbio.3000838.s008.tif]

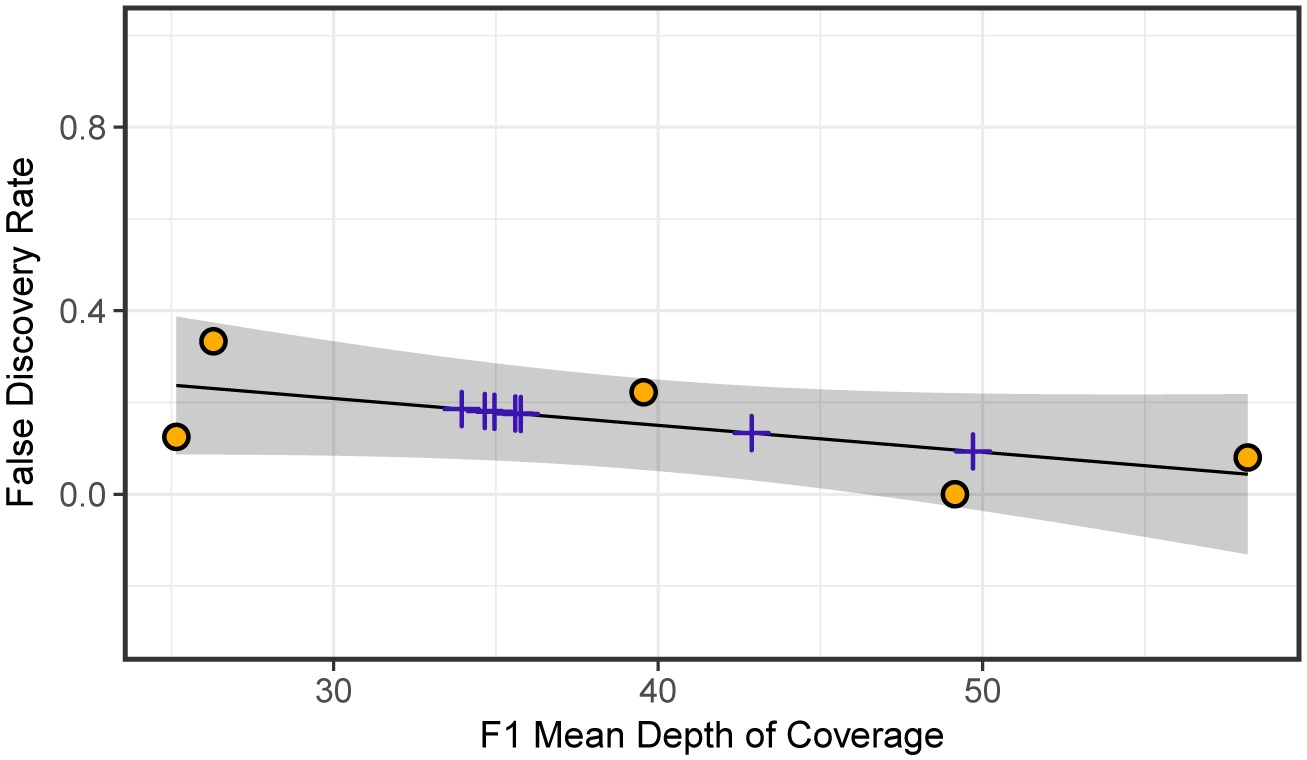

Supplement: S9 Fig — The FDR of DNM calls was inferred using a transmission-based approach in the 5 baboon F1 individuals (orange points) for which an F2 offspring was available. The black line indicates the best fit of a linear regression of these estimated FDRs against the mean depth of sequencing coverage in the same individuals. The gray-shaded region denotes the 95% CIs of the intercept and slope of the regression. The slope of the relationship is slightly negative (−0.0059) but not significantly so (p-value = 0.24). The regression fit was nonetheless used to predict FDRs in the 7 baboon F1s that lacked an F2 generation from their mean depth of coverage. Purple hash lines on the fitted line mark the predicted FDRs of these 7 baboon F1 individuals. As expected from the insignificant slope, using the mean FDR across all individuals instead does not change the qualitative conclusions and yields a very similar estimate of the mutation rate in baboons: 5.70×10^−9^ instead of 5.74×10^−9^ per bp per generation. Underlying data for this figure can be found in S2 Data. bp, base pair; DNM, de novo mutation; FDR, false discovery rate. (TIF) [file pbio.3000838.s009.tif]
