# Supplementary material for: A comparison of humans and baboons suggests germline mutation rates do not track cell divisions
Source: PLoS Biol. 2020 Aug 17;18(8):e3000838. doi: 10.1371/journal.pbio.3000838 (PMC7467331; doi:10.1371/journal.pbio.3000838)
Supplement: Attachment — Submitted filename: Response_to_reviewers.docx [file pbio.3000838.s017.docx]

Response to Reviewers

We thank the reviewers for their insightful comments, which helped us to improve the study and refine the text.

Drawing on their suggestions, we made a number of changes to our mutation-calling pipeline and reran the analysis. Notably:

- We re-called variants in baboons with the heterozygosity setting set to 0.002 (rather than the default of 0.001), which, as noted by one of the reviewers, is more appropriate for baboons. This change resulted in our detecting eight more putative mutations than previously and in a slight increase in false negative rate estimates (from 8.0% on average to 10.0%). The most marked effect it had was in uncovering new SNPs that could be used by our phasing algorithms; this resulted in an increase of our point estimate of the baboon paternal-to-maternal mutation bias, from 3.23 to 4.50 (95% CI: 2.3–13.0).
- As requested by one of the reviewers, we used the genetic map recently published by Robinson et al. (2019) to phase our baboon mutations.
- In response to the suggestion that we consider the effect of mapping errors on the false negative rate (FNR), we applied the Bamsurgeon tool to drop synthetic mutations into our read sequences and then reran the downstream pipeline. Taking into account the FNR estimated in this way, the ratio of mutation rates between the two species remains similar, although the absolute mutation rates in humans and baboons are now higher. For a number of reasons, which we discuss in the inline response, we have opted to continue reporting mutation rates estimates using our original false negative rate estimates, but have added a section to the Methods covering the effects of different FNR estimation methods.

After these changes, our general conclusions remain the same: we continue to observe an approximately two-fold lower mutation rate per generation in baboons compared to humans, and the ratio of paternal to maternal mutations remains high in both.

In rerunning the entire pipeline, we also spotted a few issues with our datasets and methods, which are now corrected, namely:

- We removed three baboons individuals that were inadvertently included in the “89 unrelated baboons” cohort (data from Robinson et al. 2019) due to a sample naming mix-up. This change resulted in our calling six additional putative mutations, which were previously filtered out incorrectly on the basis of their presence in what we thought was an entirely unrelated baboon cohort. Together with the heterozygosity adjustment detailed above, this change led to a 4% increase in the baboon mutation rate.
- A human individual that was not a member of any of the three families was erroneously included with the families when jointly calling genotypes. In order to maintain the same approach as applied to baboons, we removed the individual and reran the mutation calling and error estimation pipelines, resulting in nine additional putative mutations. These mutations had previously been filtered out on the basis of having been seen in that (unrelated) individual.
- We discovered a bug in the read-backed phasing portion of our pipeline, which led to incorrect read-backed phasing of (primarily) untransmitted DNMs. After fixing this problem, the read-backed phasing rate decreased slightly (from 36% to 32% in humans and from 58% to 51% in baboons), but when phasing was feasible by both of our methods, they remain in close agreement.

We note that in this revised version, we have also moved Laura Cox from Acknowledgements to the author list, to recognize her role in procuring baboon tissue samples, and have added a middle initial to one of the author names.

Below, please find our inline responses to the reviewers’ comments in green. Quoted text from the manuscript is *italicized*. We hope our reanalysis and revisions are satisfactory and convincing.

## REVIEWERS' COMMENTS

### Reviewer #1:

This study is interesting and adds important evidence to the ongoing puzzle of reconciling information obtained from two very different sources to the same underlying question of how mutations are generated in different species and between males and females within a species.

The major observations have been: Paternally derived mutations outnumber maternally derived mutations ~3:1 when comparing trios of mother-father-child (obs 1). On evolutionary time scales, the substitution rates present (at least) two puzzles: firstly, species which have a set of correlated traits (lower generation time, lower body mass, higher metabolic rate,...) accumulate substitutions faster (obs 2); secondly, the substitution rate is lower in more recent branches of the primate evolutionary tree than in older branches (obs 3).

A dogma in the field has been that mutations are mainly replicative in origin and that both obs 1 & 2 can be explained by the number of cell divisions leading to a germ cell.

The authors argued very convincingly against this dogma firstly in Gao et al, and now add weight, also convincingly, to that with the addition of data from baboons.

The authors also argue here that the idea of cell divisions cannot explain the differences between male rates in the two species (also convincing; a bit weaker in male/male comparison but strengthened by comparison between males and females within species)

The authors make a strong case that mutations are indeed clock-like in the two species considered; with a higher clock rate in males than in females; and maybe a higher rate per year in baboons than in humans (not totally clear on this point; please see comments below)

They also helpfully put some of the results in the wider context of other primates. It would be helpful if the data in Table 1 could be annotated with information about their relevant lineage (I had to look up whether the Owl monkey was OWM or NWM but perhaps that's just me)

We thank the reviewer for their kind words. We have included a column titled “Primate Lineage” in Table 1.

The work is sound and convincing. This is a strong paper, and my main criticism is easily addressable by a modest rewrite. I found it difficult to put the findings in the overall context of other pertinent observations regarding mutations and substitution rates in primates and the space of models that can explain the findings. The discussion is, not unreasonably, focussed on the number-of-cell-divisions hypothesis, which they have convincingly debunked. However, I think it would be good for the paper and for the field if the discussion be more forward looking. Specifically, to present how these data sit in the context of other (potentially conflicting or unexplained) findings. Specifically, does the primate data overall now (various primate pedigrees now available and reported in Table 1) suggest a stable mutation rate per year across primate species, rather than a dependence on generation time? In other words, to what extent is the generation time correlated with mutation rate per generation?

The difficulty that we face in trying to provide a more general summary of the literature is that differences in the bioinformatic pipelines and the accuracy of estimated error rates make mutation rates obtained from pedigree studies in different species largely incommensurate. As we discuss in our paper, to estimate the per generation mutation rate in baboons and humans, we try to use a single, well-calibrated pipeline in both species and check the results of our pipeline results against other large data sets in humans. Moreover, to obtain a per year estimate, we rely on our estimates of sex-specific mutation rates and on sex-specific generation times (see Amster & Sella 2016 *PNAS*). For other studies, we cannot do the same. With these limitations in mind, below is a reproduction of Table 1 to which we have added three columns: (1) A crude estimate of the mutation rate per bp per year, which we obtained by dividing the per generation mutation rate reported in the study by the average sex-averaged age of reproduction, (2) the relative yearly mutation rate ratio of the primate estimate versus our human estimate, and (3) the corresponding relative yearly substitution rate ratios in the species lineage vs the human lineage, using estimates from Moorjani et al. 2016 *PNAS*. If all these numbers are taken at face value, then our relative estimates of mutation rates in baboons and humans match the relative substitution rates, as do, perhaps, estimates in rhesus macaques, whereas in apes and Owl monkeys (a NWM), the substitution rates in the two lineages seem much more similar than do the mutation rates. We prefer not to include this information in the manuscript, because we believe it is not reliable; as just one example, a re-analysis of the same chimpanzee data in Venn et al. 2014 *Science* led to a change in the estimated mutation rate per year from 4.6×10^−10^ to 6.4×10^−10^ (Besenbacher et al. 2019 *Nat Ecol Evol*).

However, to address the more general concern that the discussion be more forward looking, we now include a discussion of how the apparent mutation slowdown in both baboons and humans (and by extension other species) could be explained, and what we think would be helpful as next steps (lines 449–468):.

*A parallel slowdown in both lineages seems highly unlikely if mutations are replicative in origin, given that changes in life history cannot plausibly explain the magnitude of the effect. In principle, one might imagine that germline mutation rates in the two species are shaped by the same exogenous mutagen and that a shift occurring after their split affected rates similarly in both lineages, leading to a parallel slowdown. If so, the change in damage rate could not have affected the ratio of male to female mutations much, since 𝛼 appears to be similar in both species. To evaluate this possibility, it will be important to obtain comparable estimates from more species, in particular an outgroup to OWM and apes such as a New World Monkey. An alternative is that the OWM fossil record has been misinterpreted to suggest a more recent split time of OWMs and apes than is truly the case.*

*More generally, while we have argued above that germline mutation patterns in humans and baboons are more easily explained if they are primarily due to damage, such a hypothesis does not provide an immediate explanation for why per year mutation and substitution rates vary across mammalian species or tend to be higher in shorter-lived ones (12–15,48). One possibility is that rates of damage co-vary somewhat with life history traits (49), with a tendency towards higher damage rates in shorter lived-species. In that regard, it would be interesting to characterize substitution rates in sets of species that differ in their environmental exposures and metabolic rates, and examine differences in their mutation spectrum in light of known mutagens (e.g., (9)).*

| **Species** | **Common name** | **Primate Lineage** | **Mean paternal age at conception** | **Sex-averaged mutation rate per generation, as reported (×10**^−^**^8^)** | **Point estimate of α, ratio of paternal to maternal DNMs** | **Number of trios** | **References** | **Mutation rate per bp per year (×10^−10^)** | **Relative yearly mutation rate (primate / human estimate)** | **Relative yearly substitution rate (Moorjani et al. 2016 *PNAS*)** |
| --- | --- | --- | --- | --- | --- | --- | --- | --- | --- | --- |
| *Papio anubis* | Baboon | OWM | 10.27 | 0.57 | 3.23 | 12 | this study | 5.49 | 1.35 | 1.35 |
| *Homo sapiens* | Human | Great ape | 34.29 | 1.23 | 3.87 | 10 | this study | 4.08 | 1.00 | 1.00 |
| *Homo sapiens (Jónsson et al.)* | Human | Great ape | 31.63 | 1.29^A^ | 4.05 | 225 | (16) |  |  |  |
| *Pan troglodytes (Besenbacher et al.)* | Chimpanzee | Great ape | 19.27 | 1.26^B^ | 4.37 | 7 | (23,48) | 7.21 | 1.77 | 1.06 |
| *Pan troglodytes (Tatsumoto et al.)* | Chimpanzee | Great ape | 24 | 1.48 | 3.08 | 1 | (47) | 6.17 | 1.51 | 1.06 |
| *Gorilla gorilla* | Gorilla | Great ape | 13.5 | 1.13^B^ | 2 | 2 | (23) | 7.64 | 1.87 | No data |
| *Pongo abelii* | Orangutan | Great ape | 31 | 1.66 | 4.13 | 1 | (23) | 7.22 | 1.77 | 1.08 |
| *Macaca mulatta* | Macaque | OWM | 7.5 | 0.37 | 1.94 | 14 | (24) | 4.93 | 1.21 | 1.37 |
| *Aotus nancymaae* | Owl Monkey | NWM | 5.55 | 0.81 | 2.09 | 14 | (22) | 12.0^D^ | 2.94 | 1.67^E^ |
| *Mus musculus* | Mouse |  | 0.44 | 0.39 | 2.76 | 40 | (4) |  |  |  |
| *Bos taurus* | Cattle |  | 5.00^C^ | 1.2 | 2.53 | 13 | (2,49,50) |  |  |  |

^A^Includes single base pair indels

^B^Mean of reported rates per individual

^C^Typical age at reproduction drawn from literature. All other ages are as reported in the relevant study.

^D^Rate estimate as reported in study.

^E^Data from marmoset, a New World Monkey.

How does the previously reported more-clock-like behaviour of CpG and and less clock-like behaviour of non-CpG mutations sit with these findings.

The behavior of CpG and non-CpG mutation rates per year was shown (but somewhat hidden, for which we apologize) in Figure S6 in the Supplement (reproduced below). We have now moved this figure to the main text as Figure 4C.

As can be seen, baboons show an almost two-fold higher yearly mutation rate at CpG sites compared to humans, despite the substitution data suggesting that they should be more clock-like. However, this comparison is complicated by the fact that CpG transitions are also subject to the effects of biased gene conversion, which may prevent the fixation of C>T mutations. As we note in the Discussion (lines 409–416):

*Since biased gene conversion on mutation acts like selection and influences the substitution process, we further broke up substitutions by type, depending on whether the fixation probability was increased, decreased or unaffected by biased gene conversion; our findings are as expected, with mutations favored (or disfavored) by biased gene conversion showing slightly higher (or lower) substitution rates relative to mutation rates (Fig. 4C). Mutation types not subject to biased gene conversion (Strong [G/C] > Strong and Weak [A/T] > Weak [A/T]) show good agreement between mutation and substitution rates.*

If the efficacy or degree of biased gene conversion were higher in baboons than humans (Li et al. 2019 *Nat Commun*), substitution rates of CpG transitions could end up being more similar between the two species than are the mutation rates.

While there appears to be no model that currently explains everything, it would be helpful to get a clear sense of which models have been down-weighted and which ones up-weighted by these new data for all of observations 1-3 above (and any other pertinent observations that I haven't listed). The authors highlight the conflict between the absolute branch lengths from substitution data and the DNM rate but is there also a conflict in the relative branch lengths?

Relative yearly mutation rates in humans and baboons are consistent with their relative branch lengths (Figure 4A–B; lines 400–404). As noted above, we have moved Figure S6 to the main text as Figure 4C to show this comparison for different mutation types.

Given these mutation rates, substitution rates suggest a split time of apes and OWMs that is implausibly old, short of a parallel slowdown in mutation rate in both lineages. We have expanded our Discussion to consider various possible explanations for our findings (lines 449–468, quoted above).

Detailed comments

1) In the introduction, various possibilities are mentioned to explain the generation time effect (pg 3, lines 70-79). it would be helpful to present the alternative explanations in the field as well (metabolic rate etc)

We have now done so (lines 80–83):

*While other correlated traits, such as body size, metabolic rate, and sperm competition, have been proposed to explain the observed dependence between reproductive span and mutation rates per unit time, in mammals, generation time remains the strongest predictor (16).*

2) pg 5, line 127. it would be worth unpacking and explaining the sentence "there should a stronger paternal age effect in baboons". there are two distinct elements to this -- both of which the authors later explore (within males comparison and male/female comparison) -- and would be helpful to explain that here.

We have revised the section to read:

*This inter-species contrast thus sets up two predictions. First, if mutations are replicative in origin and rates per cell division have not evolved, we expect fewer mutations to arise from spermatogenesis in baboons compared to humans. In other words, all else being equal, we would expect the fraction of mutations contributed by baboon fathers to be lower than that of human fathers. Second, there should be a stronger paternal age effect in baboons, in keeping with their faster rate of SSC division.*

3) I found the paragraph pg 7 lines 178-192 very difficult to understand. Please clarify. I also found it difficult to see how (in the methods) 4% FDR became 0% FDR. At any rate the sample used in sanger sequencing is not representative of the FDR per se (which is much higher in most samples) but of the approach to estimate FDR, so the FDR of this sample should not be emphasized.

The best guess of a 0% FDR estimate was obtained from our transmission-based estimation approach whereas the 4% value is the point estimate one would obtain if considering our Sanger sequencing results in isolation (1 false positive out of 24). To clarify this, we now refer to the Sanger sequencing results exclusively in terms of counts. We have also attempted to clarify the section comparing FDR approaches, quoted below:

*To measure FDRs (i.e., the proportion of spurious mutations in our putative DNM call sets), we relied on the fact that false positive DNMs identified in an F_1_ individual are very unlikely to be observed in its F_2_ offspring, whereas genuine DNMs should be observed approximately half the time. In practice, we obtained FDR estimates using a maximum likelihood based model (see Methods), which accounts for the proportion of DNMs called in both the F_1_ and F_2_ as well as the probability of observing transmission events in the first place (which is affected by the variant-calling pipeline and the sequencing coverage of the specific F_2_). We estimated FDRs separately for each trio, with median values of 21% in humans and 18% in baboons (Fig. S2B). To verify our transmission-based approach to FDR estimation, we Sanger-sequenced putative DNMs called in one human individual (both inside and outside of the orthologous regions; see Table S1 and Fig. S3). Using our transmission-based approach, we obtained a maximum likelihood estimate of the FDR of 0%, with a 95% confidence interval of 0–21%; consistent with this estimate, we found only one false positive out of the 24 DNMs that we were able to check by Sanger sequencing (p-value = 0.35, by a Fisher’s Exact Test, Fig. S4). In what follows, we used the transmission-based FDR for each trio.*

4) pg 7, lines 204, 205, please give units of age effect (DNMs per year?)

We have now done so.

5) It would be helpful to have a supplementary figure in which the data from Fig 2A and 2B are presented in the same plot (the difference in between the X and Y axes makes it harder to compare the male human/male baboon rates with female human/female baboon rates). for example, it would be helpful to see if the baboon data extrapolates into human data

We have added this figure to the Supplement (Figure S7). Please see a copy of the figure below. Focusing on plot A, although it looks as if the slope in baboon males is shallower, we are unable to reject that the slope is the same in the two species, likely for lack of power. Indeed, we do find tentative evidence that the slope in baboons differs from the one estimated for humans based on a much larger data set by Gao et al. 2019 *PNAS* (LRT p-value = 0.05).

In plot B, we have plotted paternal DNM count against the number of years between puberty and reproduction, which may be more relevant to a model in which mutations track cell divisions. Related to this plot, we can reject a model in which human and baboon fathers have the same age effect and number of DNMs at puberty in males (LRT p-value = 3.9×10^−5^). We now mention this finding in the main text as well (lines 317–320):

*The data further suggest that baboon males may have accumulated fewer DNMs by puberty than human males (LR test p-value on the intercept = 0.047) (Fig. S7) and, given limited data, are consistent with a model in which the higher intercept simply reflects the 2.4-fold older age of human males at puberty (LR test p-value = 0.12).*

6) pg 8 line 230. typo? the baboon rate is not 44% lower, it is 0.44*human rate i.e 56% lower

Thank you for catching this error, which we have fixed (using the new, somewhat revised rates).

7) pg 9, lines 237-239. please also show full analysis possibly in supplementary, not just with the subset of DNMs in F2

We have added a Figure to the Supplement, as recommended. Since this set of mutations potentially includes a substantial fraction of false positives, we expect less agreement with comparable data sets than using only mutations transmitted to F2s, and that is indeed what we see. Please see a copy of the requested figure below, and the figure with transmitted mutations (Figure 2C) below it, for comparison.

**Figure S6 (All DNMs):**

**Figure 2C (Transmitted DNMs):**

8) pg 10. line 298. typo. "humans" twice

Corrected, thank you.

9) pg 10 lines 271. is the data compatible with clock-like mutation in females i.e. is a 2.8 fold difference in the mutation rates compatible with the data

We performed a likelihood ratio test and were unable to reject a model (LR test p-value = 0.26) in which mutation rates in human mothers are 2.8-fold greater than in baboon mothers. This result has now been added to the main text (lines 306–308):

*Indeed, we fail to reject a model in which the mutation rate difference between human and baboon mothers simply reflects their typical mean ages at reproduction (LR test p-value = 0.26).*

10) pg 12 line 333. point 4. OWM mutation rates are about half that of apes: please clarify you mean per generation mutation rate

We have now clarified this point.

11) pg 12. lines 343-355. this is an important paragraph but very difficult to understand. firstly the authors state that the damage rates must be similar across mammals and the data in this paper for the two species supports that. It would be helpful to add a column in Table 1 with an estimated per year mutation rate. however, a few lines later the authors say that the "per year mutation rates vary across species and are higher in short-lived ones". this seems to be a contradictory statement. I am guessing the authors mean substitution rates in more distant species. please clarify.

Please see our response to general comments above, regarding adding a per year mutation rate column to Table 1.

We have now revised the text to read (lines 386–394):

*An alternative is that germline mutations are predominantly due to damage in both sexes. Accounting for all four observations would require damage to accrue at a relatively fixed rate across ontogenesis, but at a somewhat higher rate in males than in females, and be inefficiently repaired relative to the cell cycle length (15). In principle, this hypothesis could then explain the relative stability of the male bias in mutation in humans, the similarity of the male sex bias across mammals, and the similarity in the mutation spectrum in the two sexes. It would also explain why primate mutation rates per generation appear to roughly follow typical ages of reproduction (this paper; 23). However, it too requires a number of assumptions, and these remain to be tested.*

12) discussion final paragraph -- strong arguments that the discrepancy between the evolutionary data and present day DNM estimates cannot be explained by changes in generation times alone. Can they be explained by changes in the mutational rates per se (e.g. due to changes in the environment)? If so, please mention in discussion

We have now added a paragraph to the Discussion speculating about the possible source of the discrepancy between estimates based on substitutions and pedigree data (lines 449–468, quoted above in response to general comments).

### Reviewer #2:

Adding to the growing literature of direct mutation rate estimates in primates, Wu et al. estimate sex-specific mutation rates in baboons (Papio anubis) using resequencing data from two multi-generational pedigrees (29 individuals). Mutations either originate from errors occurring during DNA replication or from DNA damage during normal cell growth - however, the relative contribution of these mechanisms to the overall mutation rate remains unknown. As mutation rate is a quantitative trait, differences in life history traits (e.g., in physiological factors such as age) can lead to differences in rates between species. Extending their previous work in humans (Gao et al. 2016), the authors here investigate how mutations accumulate with age and cell division rates in baboons - an Old World monkey, closely related to humans, but with differences with regards to spermatogonial stem cell (SSC) division rate and mean age of reproduction. Specifically, the authors investigate whether

human and baboon mutation rates reflect differences in life history, under a model in which mutations are assumed to be proportional to cell divisions. Assuming the number of cell divisions in the germlines to be 474 in male humans, 217 in male baboons, and 31 in females (both humans and baboons), a higher male mutation bias in humans as well as a stronger paternal age effect in baboons can be expected. However, neither is observed - either, as noted by the authors, because some of their underlying assumptions are violated, or, alternatively, because a subset of germline mutations might be due to damage rather than being replicative in origin.

There are three assumptions that are critical to the study - and any violations might significantly change the expectations and interpretations of the results obtain in this study:

i) all germline mutations are replicative in origin,

ii) human and baboon ova are the product of the same number of cell divisions, and

iii) SSC division rates are 23 cell divisions per year in humans and 33 in baboons.

The assumptions raise several questions:

i) Assuming germline mutations to be replicative in origin, shouldn't the authors exclude CpG mutations from their study which are due to spontaneous deamination (i.e., non-replicative)?

As suggested, we reran two analyses after removing CpG>TpG mutations. (1) We re-estimated the paternal age effect in baboon, which is −0.52 (95% CI: −1.51–0.51), and thus still weaker compared to humans (LR test p-value = 0.012), when we would expect it to be stronger. (2) We re-estimated alpha, which remains quite similar, at 4.75 instead of 4.5, as it does in humans (Gao et al. 2019). We now make note of these findings in the main text of the manuscript:

(lines 313–316): *If anything, it appears as if paternal mutations are accruing more slowly per year in baboons than in humans (LR test p-value = 0.046), even after excluding CpG transitions, which are thought to stem mostly from spontaneous deamination (14) (LR test p-value = 0.012).*

(lines 328–334): *Under the assumptions about cell division number and rates outlined above, we would expect the ratio of male to female mutations to be roughly 2.2-fold larger in humans than in baboons (see Methods). We readily reject this model (LR test p-value = 3.84×10^−8^), even after removing transitions at CpG sites (LR test p-value = 2.30×10^−7^). Excluding these CpG transitions also yielded similar 𝛼 estimates: 4.75 in baboons (paternal fraction 83%, 95% CI: 72–93%) and 3.9 in humans (paternal fraction 80%, 95% CI: 73–85%).*

ii) Why would the same number of cell divisions be expected in human and baboon ova? And, more importantly, what are the expectations if this assumption is violated?

We agree that this assumption is tenuous and based only on the close evolutionary relationship of humans and baboons. We note in that regard that the absolute number of cell divisions experienced by mouse ova (25 c.d.) is surprisingly close to that of humans (31 c.d.) despite mice having a much shorter gestation period (Drost and Lee 1995 *Environ Mol Mutagen*). We note further that the assumption of similar numbers of cell divisions per generation is implicit in the generation time model invoked to explain variation in substitution patterns: to explain shorter lived species having more substitutions in terms of a greater number of cell divisions per unit time requires roughly similar numbers of cell divisions per generation.

Our finding of an approximately two-fold lower mutation rate in baboon females than human females per generation could potentially be explained if this assumption were wrong and if baboon ova were the product of fewer cell divisions than human ova and mutations before birth were predominantly replicative. In that case, the number of cell divisions would differ both between human and baboon sperm and between human and baboon female ova. Yet the ratio of cell divisions in males and females would somehow have to be similar in the two species, to explain why the estimated alpha is so similar. That seems coincidental, but is of course possible. More special pleading is then needed to explain why other mammals also seem to have a similar alpha, including mice (Figure 3B). Yet another complication is the presence of a maternal age effect in both humans and baboons, since these mutations cannot be driven by cell divisions. We now address these possibilities at greater length in the Discussion (lines 357–367):

*A likely possibility is that some of our assumptions are wrong. In particular, a recent review argued that germline mutations are replicative in origin, and hence track cell divisions, but that the rate of SSC divisions is much lower than previously believed (40). While plausible (40,41), this explanation alone would not explain our findings: if mutations were due to replication errors and rates of SSC divisions were very low, then without making further assumptions, we would expect human and baboon paternal mutation rates per generation to be highly similar when they are not. In turn, the approximately two-fold lower mutation rate observed in baboon compared to human females could be explained if there are fewer rounds of DNA replication in baboons than humans (or greater replication fidelity). Thus, individual observations can be explained under a replicative model by invoking specific parameters.*

iii) As noted in the Methods section, our current knowledge of germ cell division rates in baboons is limited to spermatogonial stem cells in stage 4. Given the large difference in rates observed between humans and baboons at this stage, why do the authors assume the number of cell divisions in stages 1-3 in the male baboon germline to be the same than in humans?

Only because we require a starting point to test and these assumptions give us a sensible foothold from which to work. In this case, we reasoned that the rate of cell division during the spermatogonial stem cell stage of sperm development plausibly responds to differences in mating system (this baboon species is polygynous) and levels of sperm competition between the species, so may be more malleable than earlier developmental stages; these assumptions are also widespread in previous work (e.g., Thomas et al. 2018 *Curr Biol*, Besenbacher et al. 2019 *Nat Ecol Evol*). Regardless, we fully agree with the reviewer that these assumptions are tenuous, and had already centered our Discussion on what would happen if they were not met. We have now added a more extensive discussion and changed the emphasis somewhat to make this clearer (lines 357–367; see quoted text in response to point ii).

Moreover, there are two results that warrant more detailed explanation/discussion:

i) This study did not detect any significant inter-species differences in the mutational spectra between humans and baboons. How can this observation be explained in light of Harris 2015 and Harris and Pritchard 2017 who identified both population- and species-specific mutational signatures among humans and between great apes? Additionally, in contrast to Harris 2015 and Harris and Pritchard 2017, SNPs were categorized assuming the major allele to be ancestral rather than inferring the ancestral allele using the primate multi-species alignment (which was used by the authors to identify orthologous regions).

We followed this suggestion and categorized baboon SNPs into mutation types by inferring the ancestral allele using the multialignment of baboon and rhesus macaque (*Macacca mulatta*). We additionally use the “forward variable selection procedure” implemented by Harris and Pritchard 2017 *eLife* to assess the significance of differences in mutation type frequencies between species. These changes are reflected in Figure 2C as well as in the Methods. Comparing the baboon SNPs to the Jónsson et al. dataset, we found that the proportion of T>A, C>G, CpG>TpG, and CpH>TpH mutations differ significantly between baboons and humans (p-value < 10^−4^ for all). Caveats are that the mutation spectrum seen in baboon SNPs may also reflect effects of biased gene conversion and that a proper analysis would have to account for changes in base composition between species.

Since our de novo mutation data sets are considerably smaller, we do not have power to detect these differences in the de novo mutation spectra between humans and baboons. Indeed, downsampling the baboon SNP and Jónsson et al. datasets to the sample sizes in our DNM dataset suggests that our power to detect a difference for any type is no greater than ~11% (see figure below). We now note that we likely lack power to detect the type of subtle differences reported in previous papers in the main text (lines 289–294).

ii) Assuming a molecular clock, the mutation rates reported here lead to a divergence time estimate of 63-67 million years between apes and baboons - much older than the 35 million years expected from the fossil record. Moreover, modelling changes in life history traits suggest that low generation times (3-5 years) would be required in both species to reconcile the fossil record with the estimates presented in this study. As noted by the authors, this seems rather implausibly given that "male baboons typically enter puberty around the age of six and reproduce four years later". Following Scally and Durbin (2012), the authors thus suggest a slowdown of mutation rate along the baboon lineage in order to reconcile these estimates. More discussion is needed here (indeed, it feels like the manuscript is ending rather abruptly), as the original Scally and Durbin (2012) paper argued that a hominoid slowdown, which is consistent with an increase in generation time, explains why humans have accumulated 30% fewer mutations than baboons. How would an additional slowdown in the baboon lineage fit into this? How likely is it that mutation rates have slowed in hominoids and baboons but not in other

primates? Which (potentially shared) mechanisms could even be driving these slowdowns?

As detailed in our response to Reviewer 1, we have now added a section to the Discussion (lines 449–468) speculating about why relative mutation rates in baboons and humans accord with the substitution rates, but absolute rates do not, including a parallel and proportional slowdown in both species caused by a change in an external source of damage, or an incorrect interpretation of the OWM fossil record. The former seems unlikely to us and the latter is beyond the scope of our expertise. In our opinion, reliable data from more species, notably a New World Monkey, is needed to make progress on this question.

Please see our response to general comments from Reviewer 1 and the accompanying table above for further discussion on the problems associated with fitting slowdowns to existing mutation rate estimates.

In addition to the above, there are several methodological details that warrant attention as they are likely to impact the results of this study:

i) Variant Calling: Perhaps most importantly, the authors employed a multi-sample genotype calling strategy. Han et al. (2014) have shown that multi-sample genotype calling strategies lead to an under-calling of rare variants. As DNM are by definition rare, the authors are likely to miss genuine mutations, thus underestimating rates. Unfortunately, this issue becomes worse the more data is available, thus a stronger under-calling in baboons is expected compared to humans. As this can potentially lead to severe biases in the mutation rate estimates, I would strongly encourage the authors to confirm their results using a single-sample calling strategy.

We appreciate that multi-sample genotyping calling can result in a slightly higher false negative rate than single-sample genotyping. However, it also leads to a much lower false discovery rate. Indeed, when we recalled variants in an F1 (baboon 19348) using a single-sample strategy yielded many more DNMs than the joint genotyping strategy (268 versus 34), but also a much higher FDR.

While this comparison between methods is not perfect (GATK outputs a slightly different set of quality statistics at homozygous reference sites when run in single-sample mode), it strongly suggests the loss of specificity that we expect from the single-sample calling method.

We therefore believe that the joint genotyping strategy we employ, which is the recommended strategy from the GATK Best Practices Workflow (<https://gatk.broadinstitute.org/hc/en-us/articles/360035535932>), is a reasonable approach.

Perhaps most importantly, regardless of the genotyping calling strategy, obtaining an accurate estimate of the overall germline mutation rate depends on careful estimation of false discovery and false negative rates, which is why we endeavor to obtain reliable estimates of both in this study, for each trio.

Moreover, the authors use the GATK's HaplotypeCaller with a default heterozygosity rate of 0.001, despite known higher diversity in baboons.

We are grateful to the reviewer for having spotted this important oversight, which we have now corrected. We reran our variant calling pipeline in baboons using a heterozygosity rate of 0.002 per bp, which is more appropriate for baboons (Wall et al. 2016 *Mol Ecol*; Robinson et al. 2019 *Genome Res*). After filtering, this change resulted in our identifying eight additional putative mutations.

ii) Estimating FNRs: As noted by the authors, false negatives as well as false positives can have strong effects on the estimated fraction of DNMS that are of paternal/maternal origin. Yet, the way DNMs are simulated to estimate FNR does not account for false negatives due to read mapping errors. Consequently, the reported FNR is likely an underestimate. Read processing and mapping are essential parts of the computational pipeline - with inevitably errors (e.g., due to reads mapping to locations different from the one they originated from or due to an inability to map reads against the particular reference genome). In order to incorporate false negatives due to issues with the downstream bioinformatic analyses (i.e., pre-variant calling), DNM should be simulated and put through the exact same computational pipeline than the actual data (e.g., Keightley et al. 2014).

We appreciate the reviewer’s concern, but it is not obvious how to perform these simulations realistically, and to our knowledge, the only study to do so considers simulated reads (Jónsson et al. 2017 *Nature*), which likely paint an overly rosy picture. We instead tried to assess the effect of mismapping by performing DNM simulations in both species using the Bamsurgeon “addsnv.py” tool (Ewing et al. 2015 *Nat Methods*). Bamsurgeon operates by inserting a given variant into a chosen proportion of the reads at a predetermined site and realigns the region. We applied Bamsurgeon to one human F1 and two baboon F1s. Specifically, we restricted simulated DNMs to callable, non-variable (non-SNP and non-indel) regions of our genome and randomly sampled allelic balance and alternate alleles (which means that our estimates apply only to regions with those properties). For F1s with an F2 offspring, we simulated transmission by using Bamsurgeon to insert the selected DNMs into the F2 offspring (for more detail, please see the Methods section). In total, we targeted 2000 sites for DNM simulation in F1s with an F2 offspring and 1000 sites in F1s without an F2.

One drawback of the Bamsurgeon tool is that it does not insert variants in a phase-aware manner, yet GATK Haplotypecaller builds a local haplotype graph as part of its variant-calling algorithm, and thus variants with inconsistent phase to other variant sites along the read will have their likelihoods down-weighted (Van der Auwera et al. 2013 *Curr Protoc Bioinformatics*). To guard against this problem, we created a custom script to identify simulated DNMs that failed a “three-haplotype test.” Analogously to the four-gamete test (Hudson and Kaplan 1985 *Genetics*), a DNM fails the three-haplotype test if three or more distinct haplotypes can be detected in the supporting reads. As expected, simulated DNMs that failed the three-haplotype test were filtered out by our DNM pipeline at a much higher rate than those that passed (26% versus 18% of simulated DNMs). Moreover, the proportion of variants failing the three-haplotype test was dramatically lower in the set of variants used in our original pedigree-based FNR simulation strategy compared to Bamsurgeon (5% versus 30% of simulated DNMs). To avoid these inherent issues with the Bamsurgeon tool, we focused only on the simulated DNMs that passed the three-haplotype test in the F1 as well as the F2 if one was present.

In the end, our application of Bamsurgeon resulted in FNR point estimates of 19.2% for the human F1 and 16.5% and 17.7% for the two baboons. Since the increase in FNR between the two strategies is similar in both species, the baboon-to-human mutation rate ratio remains consistent across different FNR strategies (0.47 using our previous approach vs 0.46 using Bamsurgeon). At the same time, because the FNR rate is ~1.8-fold higher than we had estimated in our pedigree simulations, the absolute mutation rates are now roughly 8% higher in each species.

We note, however, that while poorly mapped reads can indeed be aligned away from their true origin, causing one to miss the DNM and generating a false negative, mis-alignment could also create one or more spurious DNMs—false positives—in the mismapped region. Thus, it seems that mapping errors, at least in principle, can manifest as both missed calls (false negatives) and false discovery events, and we are only modeling the former by using Bamsurgeon. Moreover, we cannot place full confidence in our Bamsurgeon results since, as we demonstrated, the tool is not phase or LD-aware. Bamsurgeon was designed to simulate somatic mutations and as far as we know has not been validated for germline mutations. Only one germline mutation study of which we are aware (in herrings; Feng et al. 2017 *eLife*) compared synthetic mutation insertion against a pedigree method like ours and they found a lower FNR when inserting in silico mutations into reads, opposite to our findings.

Given these considerations, we have opted to focus on absolute rate estimates that incorporate our original pedigree-simulation FNR estimates. However, we discuss the effects of different DNM simulation methods—including the results presented above—in the Methods (lines 776–843). We now explicitly note the effects of read mapping errors in the Results and that our FNRs are likely somewhat under-estimated (lines 202-206).

Moreover, with regards to the read alignment, repeat regions are well-known for causing mapping issues. As repeats have not been excluded in this study, could the authors please comment on how many DNM were observed in repeat vs non-repeat regions of the genome?

We observe a 26% increase in mutation rate in repetitive versus non-repetitive regions in humans and a 63% increase in baboons.

We have added a supplementary figure (Figure S5B reproduced above) to report mutation rate variation in different compartments of the genome. As can be seen, in orthologous regions, there is little difference; in contrast, mutation rates are significantly higher in non-orthologous, repetitive regions than in non-orthologous, non-repetitive regions (Poisson rate ratio test, p‑value < 10^−3^ for both species). An increase in mutation rate in repetitive elements genome-wide has been observed in earlier, larger studies, in humans (Turner et al. 2017 *Cell*). One interpretation is that repetitive regions have a higher mutation rate; another is that the apparent increase in the mutation rate is due to mapping errors in repetitive regions that cannot be aligned between species. We have added these results to our manuscript (lines 251–258).

iii) Identification of DNMs: The authors require DNMs to be supported by at least 3 alternate reads in an offspring. DNMs are then classified as "transmitted" to the F2 if at least 2 reads support the DNM. It is unclear why the same read thresholds are being used, given the vastly different read coverage in the species (i.e., 24X in humans vs. 45X in baboons on average). To allow for fairer comparisons, the same percentage (rather than count) of alternate reads should be utilized.

We respectfully disagree. The alternate read filter is aimed at eliminating chance sequencing errors in regions of especially low coverage. Since the sequencing error rate is on the order of 1%, we require the variant to have been seen at least three times in the F1, to ensure that the random error rate is on the order of 10^-6^ or less. (Since we only use the F2 to verify transmission of putative mutations, we don’t need to be that strigent, so relax it to an error rate of 10^-4^.) This alternate read filter in F1s is applied *in addition to* the allelic balance filter, which takes the total depth of coverage at the site into account. Most importantly, we estimate the false negative rates in each species separately, and those false negative rates account for our precise criteria and differences in coverage.

iv) Male-to-Female Mutation Ratios: The authors state a lack of a fine-scale genetic map for baboons when estimating male-to-female mutation ratios. However, a fine-scale LD-based map is available from Robinson et al. 2019 and should indeed be used rather than assuming an (unrealistic) uniform map.

Following this suggestion, we updated our baboon analysis to use the Robinson et al. 2019 *Genome Res* genetic map. To this end, we converted the LDHelmet recombination map from Robinson et al. 2019 to a genetic distance map, using a method described by Booker, Ness, and Keightley 2017 *Genetics* and the chromosomal map lengths measured by Rogers et al. 2000 *Genomics*. The procedure is described in the Methods section (lines 877–885). We found no difference in the inferred phasing of the DNMs, likely due to the fact that the map acts as a prior in the SHAPEIT algorithm and the phasing results are primarily informed by the data. We also used this map to generate confidence intervals around certain estimates by bootstrap resampling of 50 cM blocks; again, none of the results changed.

v) Base Quality Score Recalibration: The authors note that there is no "gold standard" dataset available for Base Quality Score Recalibration in baboons. Robinson et al. 2019 published a variant dataset containing >50 million SNPs in 100 baboons that could be utilized here. Perhaps this is the second dataset from the sequencing panel of 89 unrelated baboons that the authors are referring to in their Methods section (please note that the cited paper, reference 49, is pointing to a study in cattle)? If it indeed is the same dataset, what are the differences in filtering strategy applied by the original work and this study?

The sequencing panel used in this step is indeed the Robinson et al. 2019 *Genome Res* dataset. By a “gold standard” SNP set, we mean that, unlike in humans, there is not yet an agreed-upon set of baboon sites that are known to be commonly variable (e.g., having been called by multiple independent sequencing approaches). We have changed the text and the citation to more accurately reflect what we mean (lines 634–636). The filtering strategy used by Robinson et al. bears some similarities to our study (e.g., we use the same “hard filter” cutoffs). However, since our end goals differ—DNM calling rather than SNP calling—the particular cutoffs of certain filters (e.g., genotype qualities) are inevitably different, with ours being generally more stringent.

vi) The authors state that the application of different computational pipelines hinders the comparison of mutation rate estimates among primates. While I completely agree with this statement, the differences in the experimental design might also introduce biases. In this study, 100bp paired-end Illumina HiSeq 2000 sequencing was used for human individuals while 150bp paired-end Illumina HiSeq X sequencing was used for baboon individuals. There are two points of importance: i) Heng Li (and others) have previously noted that empirical error rates are higher in HiSeq X than in HiSeq 2000 and ii) longer reads usually align more reliably than shorter reads. Neither has been taken into account in the presented analyses (or, potential implications discussed). In addition, mean coverage differs markedly between the species (i.e., 24X in humans vs. 45X in baboons).

We agree that the difference in the sequencing platforms or protocol is a limitation of our study, which may influence the resulting mutation rates, and now make explicit mention of this fact in the Methods (lines 609–614):

*We next set out to identify DNMs and estimate trio-specific error rates in a similar manner across both baboons and humans with the aim of minimizing the technical biases that can confound interspecies comparisons. We note, however, that systematic differences between the two Illumina platforms used for sequencing—while largely unavoidable—could potentially influence our final mutation rates.*

However, we have constructed our pipelines and our comparisons to be as similar as possible in all other regards and, most importantly, we have estimated trio-specific (and hence species specific) false discovery and false negative rates. We would argue further that if the sequencing platform had a qualitative effect, then these comparisons are rather hopeless, as such differences are present across most non-human samples (as one example, Besenbacher et al. 2019 *Nat Ecol Evol* combines Illumina HiSeq X and HiSeq 2000 sequences) and are inevitable due to the rapid turnover of sequencing platform offerings at sequencing cores year to year. Reassuringly, however, that does not appear to be the case, given that groups such as Decode have obtained largely consistent results on a variety of different sequencers over the past decade (Kong et al. 2012 *Nature*, Jónsson et al. 2017 *Nature*, Halldorsson et al. 2019 *Science*). In our opinion, a bigger concern are differences in the quality of the baboon and human reference genomes, which is why we focus on orthologous regions and try to estimate species-specific (and in fact trio-specific) error rates.

vii) Data Access: No code has been shared with the reviewers hindering a more thorough evaluation of the results.

We apologize. We had always planned to make code available, as is the practice of the lab (see <https://przeworskilab.com/data/>) and would have been happy to provide them to the reviewer; we were waiting to revise our pipeline in light of the reviewer comments, as has happened. Code for reproducing the figures and numbers reported in the manuscript is now available on GitHub at https://github.com/flw88/baboon_dnms .

Lastly, some minor comments:

* Sex-averaged germline mutation rate estimates for humans range from 1.25 x 10-8 to 1.34 x 10-8 per base pair per generation in orthologous and all callable regions, respectively. A similar pattern is seen in baboons. Is there any explanation for the lower mutation rate estimates when only orthologous regions are considered?

We have added an analysis of mutation rate variation across repetitive and orthologous regions of the genome. We find that elevated mutation rates in non-orthologous regions of the genome appear to be be driven by higher rates in repetitive regions. Please see our discussion above in response to comment (ii) part two (Reviewer 2).

* Estimates from Tatsumoto et al (2017) for chimpanzees were excluded from the comparative analysis. As Tatsumoto et al's estimates differ from those reported by Besenbacher et al. (2019), it would be interesting to check whether both agree with regards to the analyses presented here.

As demonstrated in the table below, there is little difference whether the Besenbacher or Tatsumoto chimpanzee estimates are excluded from the linear regression of proportion paternal DNMs versus paternal age.

| **Excluded estimate** | **Intercept est.**  **(95% CI)** | **Intercept**  **p-value** | **Slope est.**  **(95% CI)** | **Slope**  **p-value** |
| --- | --- | --- | --- | --- |
| Tatsumoto et al. (2017) | 0.70  (0.62–0.77) | 8×10^−8^ | 3.4×10^−3^  (−0.6–7.4×10^−3^) | 0.09 |
| Besenbacher et al. (2019) | 0.69  (0.63–0.76) | 5×10^−8^ | 3.0×10^-3^  (−0.6–6.6×10^−3^) | 0.09 |

* line 533 states that 26 baboons were used in this study while other parts of the manuscript state 29 individuals.

Corrected, thank you.

### Reviewer #3:

This paper presents an interesting and carefully conducted study of de novo mutation rates measured in humans and baboons. The authors find that baboon mutation rates per generation are approximately half that of humans, largely because of their lower generation time, and that consistency with their phylogenetic divergence seems to require a mutational slowdown in not just humans both both species. The ratio of maternal to paternal mutations is nearly the same in both species and inconsistent with the hypothesis of a replicative origin of most de novo mutations, adding additional evidence to a hypothesis that the authors previously published in an analysis of human data. Although I have a few suggestions for improving the manuscript, they are quite minor and are not make-or-break issues.

1. The authors specify that the mutation rates of the aligned callable genomic regions are slightly lower than the mutation rates of the entire callable genomic regions in each species. Although the magnitude of the difference is low, it is potentially significant if it means that the mutation rate of the currently callable genome might not be representative of the rest of the genome. Can the authors specify whether the mutation rates of the homologous regions and nonhomologous regions are significantly different, and whether any significant difference can be explained by differences in repeat content? Is mutation rate symmetrically elevated in the nonhomologous regions from both species? This could be important if it lends credence to the idea that human and baboon DNA might have mutated faster at an earlier point when it perhaps had higher repeat content or was otherwise functionally different than it is now.

Please see our response to comment (ii) of Reviewer 2, who raised similar points.

2. Unless I missed it, the manuscript doesn't test whether there's a significant difference between humans and baboons in the number of additional mutations accumulated per year of parental age or the y-intercept expected number of mutations already accumulated by puberty. It would be interesting to specify these to give readers a sense of which of these values appear static vs evolutionarily labile over these few million years of divergence.

We did not find a significant difference (LR test p-value = 0.19) between paternal age effects in humans and baboons but only briefly alluded to this result in the legend of Figure 2. To clarify these findings, we have expanded the analysis to include a comparison of the y-intercept at puberty and have added a figure to the Supplement (Figure S7) in which both species are plotted together so that age effects and intercepts at puberty can be visually compared. Please see a reproduction of this figure under comment 5 of Reviewer 1, who raised a similar point. We have also added a brief paragraph in the Results section, in which we discuss the comparison of the y-intercept at puberty (lines 317–321).

3. From Figure 1, it looks like paternal age might explain less of the variation in mutation rate between baboon families than between human families. Is this an accurate assessment, or is the excess dispersion of the baboon data points simply the result of lower mutation counts and higher noise?

We thank the reviewer for this suggestion. To look into this question, we fit a negative binomial regression to the paternal mutation data in each species. Whereas the the Poisson model fixes the variance to be equal to the mean ($\text{E}\left[ Y \right]=\text{Var}\left[ Y \right]=\mu$), the negative binomial model allows for an additional source of variation ($\text{E}\left[ Y \right]=\mu; \text{Var}\left[ Y \right]=\mu+\mu^{2}/\theta$). Performing a likelihood ratio test with the Poisson as a null model, we found a significant p-value in humans (p-value = 0.017, $\theta\approx$ 40) but not in baboons (p-value = 0.30, $\theta\approx$ 49); in other words, we only find evidence for excess dispersion in humans (as also found in Gao et al. 2019 *PNAS*).

Additional line by line comments:

Line 119 states that baboons enter puberty "around age 6 and reproduce four years later." Does this mean that age 10 is the mean age of first reproduction, or the mean age of a randomly sampled baboon's parent?

We apologize for the confusion. 10 is the mean age of a randomly sampled baboon’s parent. We have revised the text to clarify this point.

Line 280 states that "paternal mutations are accruing no faster with age in baboons than humans (LR test p-value = 0.045)." Doesn't the fact that this p-value is less than 0.05 imply a marginally significant difference, not a lack of difference?

We have reworded the text accordingly.
