# Supplementary material for: A comparison of humans and baboons suggests germline mutation rates do not track cell divisions
Source: PLoS Biol. 2020 Aug 17;18(8):e3000838. doi: 10.1371/journal.pbio.3000838 (PMC7467331; doi:10.1371/journal.pbio.3000838)
Supplement: Attachment — Submitted filename: Response_to_reviewers.revision2.docx [file pbio.3000838.s018.docx]

Response to Reviewers

Please find our inline responses to Reviewer #2’s comments below, in green. We hope our revisions are now satisfactory.

REVIEWERS' COMMENTS:

Reviewer #1:

I am satisfied with the revisions and have no further comments or queries.

Reviewer #2:

The authors have addressed the majority of my previous concerns in the revised version of their manuscript though please note that reviewers are still unable to access the code to evaluate the results as the GitHub link results in a 404 error (see my previous comment regarding Data Access). Overall, this paper adds nicely to the growing literature of DNM estimation in primates.

We sincerely apologize: the GitHub repository sharing settings were not properly configured to “public.” We have corrected this issue and the URL provided in the text (<https://github.com/flw88/baboon_dnms>) should now link properly to the repository. Please let us know if there is any problem accessing the website.

I only have a few minor comments on the revised version of the manuscript:

The authors mention several DNM studies in the main text that are absent from Table 1 (e.g., a direct mutation rate estimate for chimpanzees (Venn et al. 2014), green monkeys (Pfeifer 2017), and wolves (Koch et al. 2019)). To allow the reader to navigate and compare the current (and still limited) literature on the topic more easily, these should be added to Table 1. In addition, Table 1 in the response to the reviewer comments has two additional remarks (D: Rate estimate as reported in study and E: Data from marmoset, a New World Monkey.) that are missing from Table 1 in the manuscript.

We apologize for the lack of clarity. In Table 1, we intentionally included only those mammalian species for which pedigree estimates of *sex-specific* mutation rates are available, given that we were interested in comparing the degree of male mutation bias across mammalia. The wolf and green monkey studies (Koch et al. 2019; Pfeifer 2017) do not provide estimates for $\alpha$ or phased de novo mutation data, which is why we did not include these studies from the table. In addition, the chimpanzee data from Venn et al. 2014 was fully re-analyzed by Besenbacher et al. 2019, so we reported the Besenbacher et al. results; we note that Venn et al. are cited in the “References” column. To clarify our selection criteria, we have added a title, legend, and footnote to Table 1.

Regarding footnotes D and E in the Response to Reviewers table, these remarks were included as part of three columns that were added in order to address questions from Reviewer #1 concerning the overall picture of yearly mutation and substitution rate variation across primate species (column names: Mutation rate per bp per year; Relative yearly mutation rate; Relative yearly substitution rate). As we noted in our Response, however, we prefer not to include this information in the manuscript, given that “differences in the bioinformatic pipelines and the accuracy of estimated error rates make mutation rates obtained from pedigree studies in different species largely incommensurate.” Another consideration in that regard is that we relied on our sex-specific mutation rate estimates and on sex-specific generation times to calculate yearly mutation rates (see Amster & Sella, 2016), which we cannot readily do for other studies. For these reasons, we chose not to include these additional columns—and by extension footnotes D and E—in Table 1.

Early studies in humans were unable to detect the more subtle maternal age effect and only with subsequent sequencing of hundreds to thousands of trios such an effect was detected in the species. In this regard, it is remarkable that the authors were able to discern a maternal age effect in baboons with merely two pedigrees/29 individuals. Could the authors please comment on why, in their opinion, no maternal age effect has been reported in similarly powered studies of other non-human primates (e.g., Thomas et al. 2018; Besenbacher et al. 2019)?

That is a very good question, to which we don’t have an answer. One possibility is that we have slightly more power to detect maternal effects than previous studies, as the power depends not only on the number of trios but also on the number of reliably phased mutations. In baboons, we were able to phase 151 of 248 of the DNMs, a slightly higher number than in Thomas et al. in macaque (105 out of 283) or Besenbacher et al. in chimpanzee (102 out of 263). Another possibility is that the maternal age effect in baboons is somewhat stronger than in other non-human primates. Yet another is that it is a statistical fluke (our p-value is not that low, at 0.043, and the exclusion of one point suffices to raise it to 0.196). We have now added a sentence of caution about this result (lines 302–304):

*“Intriguingly, we obtained a significant maternal age effect of 0.55 DNMs per year (95% CI: 0.03–1.02; p-value = 0.043); however, the p-value for the maternal age effect is strongly dependent on the inclusion of one somewhat outlier point.”*

lines 272 and 273: "To understand how the distribution of single base pair mutation types (i.e., the mutation "spectra") (…)"

It should be "distribution and mutation spectrum" (singular) or "distributions and mutation spectra" (plural).

Corrected, thank you.

Reviewer #3:

In my opinion, the authors have responded satisfactorily to all comments.
